# Atom-by-Atom Iterative Synthetic Logic: Laying the Foundation for Programmable Automated Construction of Small Organic Molecules

**DOI:** 10.1021/acscentsci.5c00526

**Published:** 2025-05-29

**Authors:** Miao Chen, Guangbin Dong

**Affiliations:** Department of Chemistry, 2462 University of Chicago, Chicago, Illinois 60637, United States

## Abstract

Fully automated preparation of diverse small organic molecules remains a formidable challenge due to the inherent constraints of conventional synthetic philosophies. The existing automation approaches require access to either almost unlimited kinds of chemical reagents or custom-made building blocks (BBs). Herein we propose atom-by-atom iterative synthesis (AIS) as a new synthetic logic to tackle this challenge. By viewing complex organic molecules as assemblies of single-carbon- or heteroatom-based units, AIS aims to construct molecular skeletons through iterative coupling of simple atomic-scale BBs by a unified type of reaction—boron homologations. Compared with conventional approaches, the AIS strategy uses only a few types of chemical reactions and a small set of BBs, making it more suitable for automation and artificial intelligence-assisted synthetic route design. To date, enormous progresses have been made on the synthetic chemistry that serves for the purpose of AIS, such as introducing heteroatoms and sp^2^-carbons, forming ring structures, developing thermostable carbenoid reagents, and achieving stereochemical controls. On the other hand, substantial challenges and limitations remain to be overcome for realizing fully automated construction of diverse molecules. This Outlook article describes the AIS concept, recent progress, current limitations, and future opportunities in this field.

## Graphical abstract

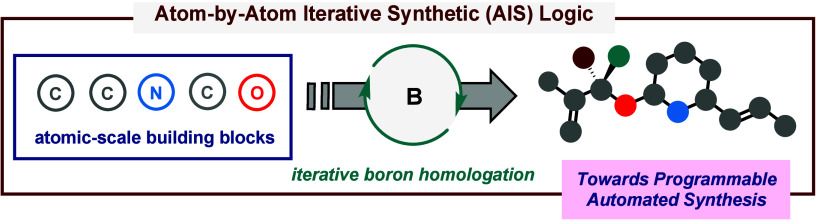

## 1 Introduction

The past two centuries have witnessed transformative changes to the human society enabled by a series of industrial revolutions, which have shaped our economies, cultures, and individual lives.^1,2^ For instance, smart automation and artificial intelligence (AI), featuring in the fourth industrial resolution, have significantly improved production and work efficiency across various sectors.^3–5^ However, the adoption of automation in synthetic chemistry operations, particularly in preparing fine small organic molecules, has lagged far behind other fields.^6^

The adoption of automation in synthetic chemistry operations, particularly in preparing fine small organic molecules, has lagged far behind other fields.

Small organic molecules play critical roles in our daily life by serving as drugs, agrochemicals, materials, fuels, health supplements, cosmetics, food additives, and household chemicals. New functional molecules are constantly needed for various purposes; however, their preparation is often time-consuming and requires special expertise. For example, it takes medicinal chemists months to access a new molecular scaffold of medium complexity, and process chemists 1–2 years to optimize a scale-up. In academia, completing a total synthesis project often requires two to three years for a medium-size natural product and even a decade for more complex ones. Although our synthetic capability has increased dramatically, time and cost continue to limit our capacity. In addition, it remains extremely challenging or costly for researchers who are not synthetic chemists (e.g., those in other fields) to access noncommercially available organic molecules. Thus, the demand for fully automated organic synthesis has become increasingly evident.^7–9^

The automated technologies to access oligonucleotides and peptides have made a game-changing impact on advancing molecular biology and developing biologic drugs. However, organic compounds present many more challenges to realize automated preparation. This is because, unlike peptides/oligonucleotides, regular organic molecules lack repeating patterns, linear shapes, and uniform linkages; instead, they exhibit more diverse structures. The conventional tactics for synthesizing small organic molecules are primarily based on functional group (FG)-guided fragment assembly (FGFA), which leverages reactivity of different FGs to assemble commercially available chemicals into fragments that are then assembled into target compounds.^10^ It is clear that the FGFA approach is centered on the FG transformations. Owing to the wide range of efficient synthetic methods on FG transformations that have flourished in the past century, the FGFA approach has found vast success in constructing complex organic molecules. A representative example is shown in the recent total synthesis of bryostatin 3 by Trost^11^ (Figure 1A). The complex molecule was elegantly put together from three fragments in a convergent manner, and each fragment was efficiently synthesized from commercially available materials. In this total synthesis, 27 types of reactions were employed, and ∼60 kinds of reagents/reactants (without counting solvents) were needed.

**Figure 1:**
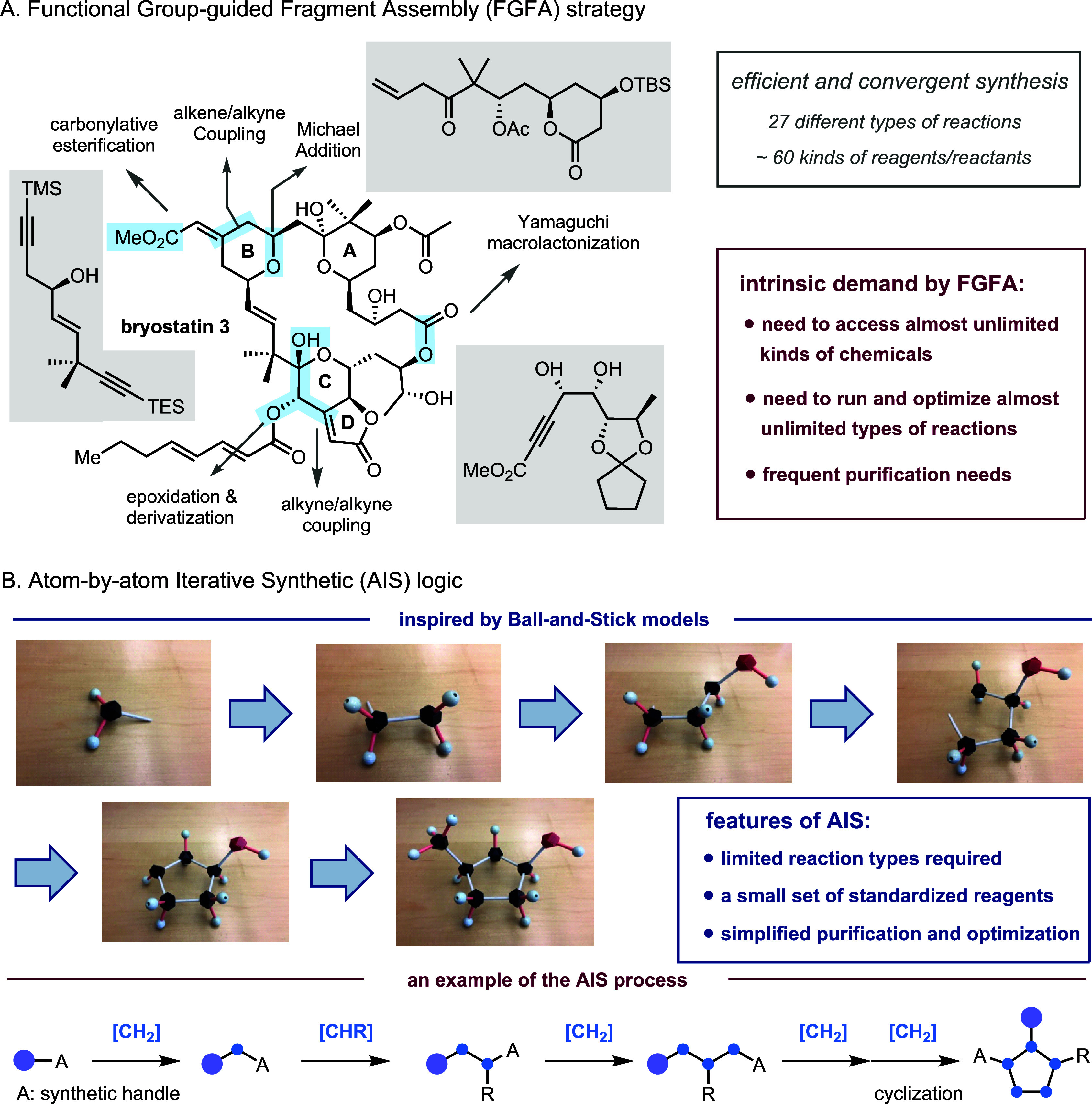
Functional group (FG)-guided fragment assembly (FGFA) versus atom-by-atom iterative synthetic (AIS) logic. A, a representative example of the traditional FGFA strategy. B, conceptual depiction of the AIS logic inspired by plastic molecular models.

One can imagine that an automated synthesizer that relies on the FGFA approach would need to be able to access almost unlimited kinds of chemicals, to carry out and optimize almost unlimited types of reactions, and to handle purification of compounds with various properties in order to construct diverse organic molecules. These intrinsic demands would add substantial challenges to the development of a *compact* and *versatile* automated synthesizer, even with the aid of advance technologies, such as AI-enabled retrosynthetic design,^12,13^ integrated modular flow platforms,^6^ or advanced robotic systems.^14–17^

One key factor for the wide success of automated syntheses of oligonucleotides and peptides is the use of an iterative coupling strategy, which allows for simplifying the synthetic process by focusing on just a few types of reactions.^18–21^ It is noteworthy that by taking advantage of iterative Suzuki coupling, in 2015 Burke and co-workers realized an impressive automated small-molecule synthesizer using bifunctional building blocks (BBs) that contain a halogen group and a protected boronate.^22–27^ The Burke system has been successfully integrated with flow chemistry and found attractive utility in material discovery. Nonetheless, the BBs employed in this system are relatively larger pieces and typically require a custom design.

Clearly, the use of simpler and more available BBs would be more attractive for an on-demand automated molecular synthesizer; however, the structural diversity of small organic molecules poses substantial challenges for adapting iterative synthetic strategies. Here, we are inspired by a fact that a plastic molecular model set only contains about a dozen types of BBs, but it can build almost unlimited kinds of molecular structures (Figure 1B). In this process, complex molecules are constructed *atom by atom*. It becomes obvious that smaller BBs are generally more versatile and adaptable for creating more diverse structures. A few intriguing questions then arise: Can backbones of most organic molecules be broken down into one-carbon or one-heteroatom units? Can these small units be connected one by one in a controlled manner? If the prior two questions can be addressed, can diverse organic compounds then be prepared from a small set of simple BBs through iterative couplings? Moreover, unlike the more convergent FGFA strategies, one can imagine that atom-by-atom assembly is an inherently linear process, demanding high-yielding reactions in each step. Thus, can robust, versatile, and exceptionally efficient coupling strategies be developed? Based on these questions, this Outlook article aims to offer a vision on the **a**tom-by-atom **i**terative **s**ynthetic (AIS) logic, which is to *construct organic molecules through iterative assembly of atomic-scale BBs*, and an overview of the recent progress. The objective here is by no means to be comprehensive, but instead, we hope to show the promises and potential challenges of AIS for enabling programmable automated construction of small organic molecules.

We are inspired by a fact that a plastic molecular model set only contains about a dozen types of BBs, but it can build almost unlimited kinds of molecular structures.

## 2 Discussion

### 2.1 Concept and General Considerations

Based on the AIS logic, we hypothesized that most organic molecules could be broken down into one-carbon or one-heteroatom units; if these small units can be connected one by one in a controlled order, assorted organic compounds could then be prepared from a small set of simple BBs.^28^ The key for the AIS strategy is to identify one kind of robust and high-yielding reaction to connect BBs and to employ atomic scale reagents, such as carbenoids (divalent carbons) and similar species (e.g., nitrenoid or oxygen), as BBs. To allow access to diverse scaffolds with a limited variety of BBs by AIS, the iterative reactions employed must exhibit the following features: 1) the reaction needs to be general, meaning that diverse substrates and BBs could all react; 2) the reaction needs to be chemoselective, meaning that the reactive terminus should react much faster than other FGs; 3) the chain propagation needs to be controllable, meaning that only one BB is added at the reactive terminus at one time; 4) the BBs or monomers used in the reaction need to be simple and easily accessible. Based on these considerations, the Matteson-type homologation could meet all these criteria and serve as a promising reaction to connect BBs (Figure 2A).^29–33^ The conventional Matteson reaction involves boron-based 1,2-metalate migration that enables one-carbon homologation of boronates. It uses an electropositive boron center as the reactive terminus or synthetic handle (e.g., **A** group in Figure 1B), which chemoselectively reacts with an organometallic nucleophile that contains a leaving group (LG: X), namely a carbenoid. The borate adduct is stable at low temperatures, while at higher temperatures, 1,2-migration takes place to form the C–C bond and regenerate the reactive boron terminus that can undergo further homologations. The 1,2-migration is typically stereospecific because the σ* orbital of the C–X bond needs to align with the σ bonding orbital of the C–B bond in this reaction. The overall process could be viewed as a formal carbene insertion into the C–B bond.

**Figure 2:**
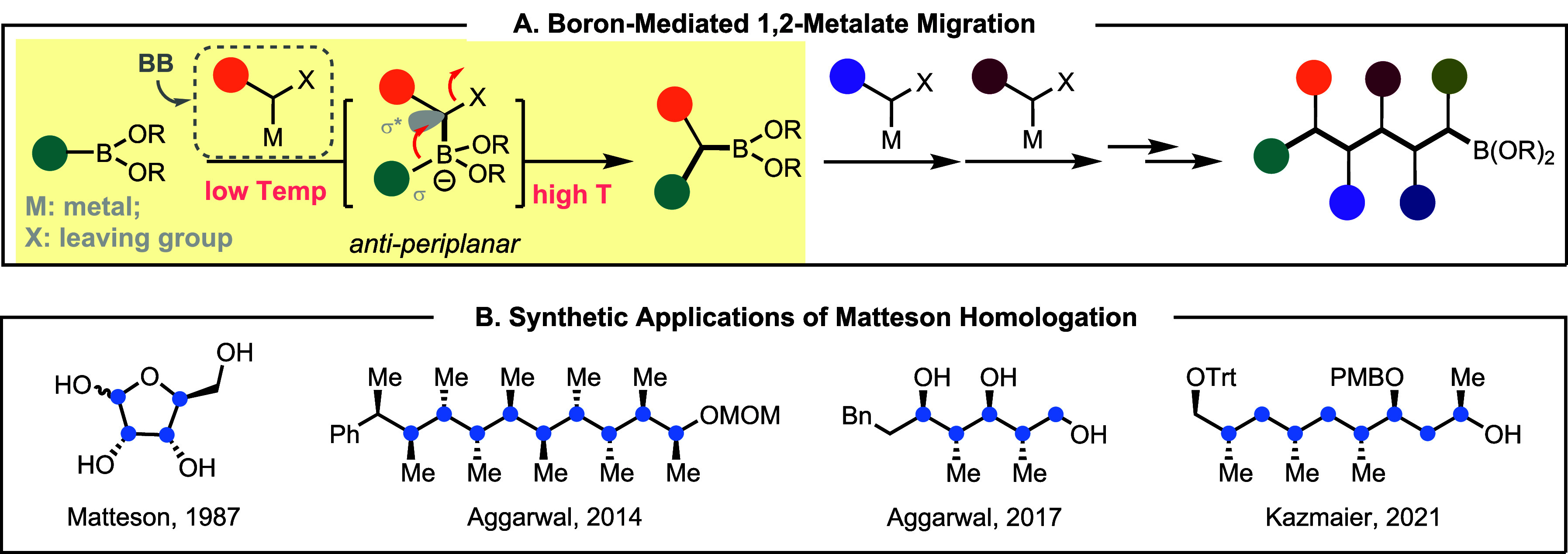
Key chemical reaction for connecting building blocks (BBs) in AIS. A, the general mechanism of Matteson homologation and the use in programmable synthesis. B, representative synthetic applications of Matteson homologation in constructing linear carbon chains with defined stereochemistry.

This Outlook article aims to offer a vision on the atom-by-atom iterative synthetic (AIS) logic, which is to construct organic molecules through iterative assembly of atomic-scale BBs, and an overview of the recent progress.

The use of Matteson homologation, including Aggarwal’s variation in assembly line synthesis, has been elegantly demonstrated in the construction of simple linear compounds based on sp^3^-carbons or a linear fragment of natural products (Figure 2B).^34–40^ However, most organic compounds feature more than just sp^3^-hybridized carbons or linear frameworks in their structures. Therefore, a number of key questions need to be addressed in order to make Matteson-type homologation suitable for the automated synthesis: *(a) How to introduce heteroatoms, such as O, N, or S atoms, into the molecular skeletons? (b) How do we construct ring (2D) or cage (3D) structures atom-by-atom? (c) How to deal with branched structures, as well as non-sp* ^3^ *units? (d) How do we install sensitive FGs as substituents? (e) How to enable practical and robust stereochemical control? (f) How to reach high (>95%) yield in each step to simplify purification? and (h) How to determine the optimal synthetic route when there are so many possibilities?* The following subsections summarize recent progresses toward some of these questions.

#### 2.1.1 Heteroatom Insertion

The Matteson reaction has been extensively developed for the homologation of a sp^3^-carbon unit. However, to realize automated organic synthesis, it becomes important to enable introduction of heteroatoms, such as N, O or S, into the molecular backbones and to ensure the continued chain homologation. Clearly, the hetero-Matteson reactions are essential for the proposed automated synthesis, which would require the development of suitable heteroatom BBs.

Inspired by the seminal works in organoborane chemistry,^41–49^ the recent studies by the Dong group have allowed iterative insertion of nitrogen and oxygen atoms during the chain homologation of boronates (Figure 3).^50–52^ In 2021, they developed the aza-Matteson reaction by enabling controlled mono- and double-methylene insertions into N–B bonds. The insertion selectivity highly depends on the LG in the carbenoid.^50^ When (bromomethyl)lithium is used as the carbenoid, the 1,2-metalate rearrangement can occur at a very low temperature (−78 °C) as bromide is an excellent LG. As such, the initially formed monoinsertion product reacts with the carbenoid again to form a more stable ate complex at the low temperature, which is later converted to the double methylene insertion product after warming the reaction up to a higher temperature. In contrast, the use of the chloride-based carbenoids forms a stable ate complex at the low temperature, which leads to mono methylene insertion. After the development of C- insertion into N–B bonds, they further discovered a suitable class of nitrenoids containing a carbamate LG to allow N-insertion into C–B bonds.^51^ Consequently, the iterative nitrenoid and carbenoid insertions into boronates enabled the programmable synthesis of amines.

**Figure 3:**
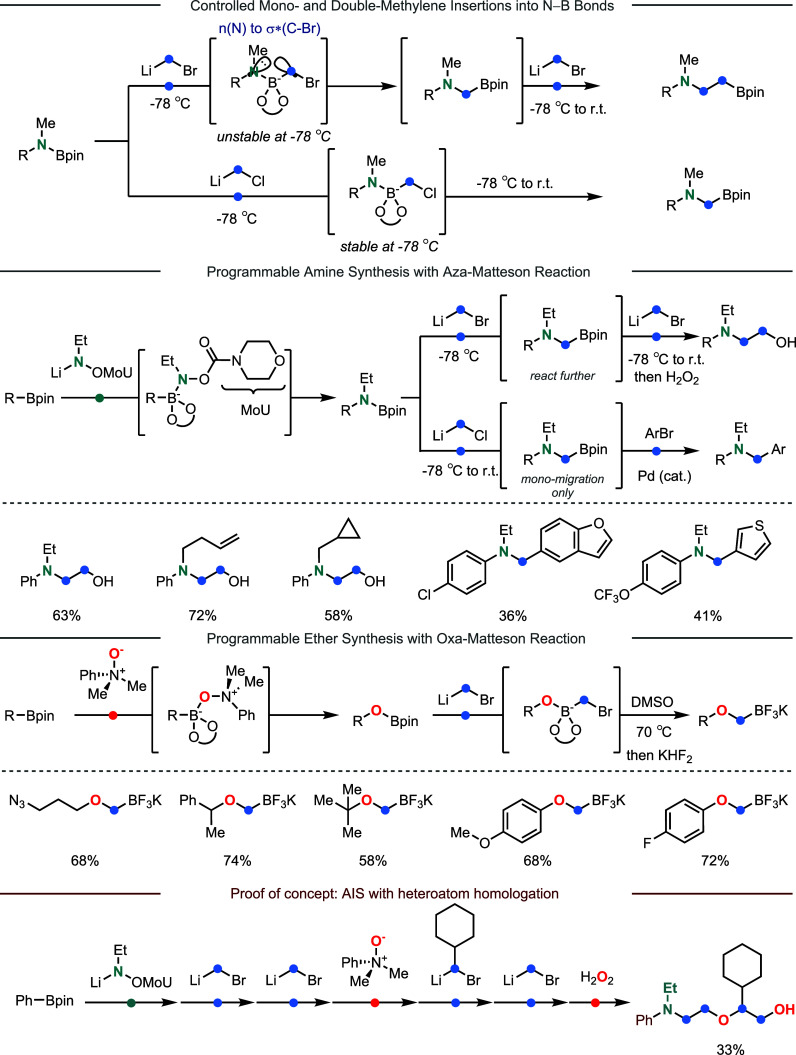
Development of boronate homologations based on heteroatom insertions.

In 2022, the Dong group extended their study to allow incorporation of oxygen atoms.^52^ The oxa-Matteson reaction employed a tertiary amine oxide as the oxygen-BB, which mediates an initial O-insertion—conversion of a C–B bond into a C–O bond—followed by carbenoid insertion into the resulting O–B bond to yield the corresponding boron-substituted ethers. The broad substrate scope and tolerance of various alkyl and aryl boronates along with a wide array of FGs underscore the versatility of this method for synthesizing complex ether structures. To validate the concept of AIS, a structurally complex molecule containing multiple heteroatoms was successfully synthesized in 33% overall yield through programmable insertion of N/O/C-based BBs.

#### 2.1.2 sp^2^-Carbon Insertion

π-Bonds are ubiquitously found in organic molecules, but conventional Matteson reactions only form σ bonds. Thus, a key question is whether simple strategies can be realized to access structures containing non-sp^3^ groups via iterative boron homologation. In 2019, Morken and co-workers introduced a Pd-catalyzed 1,2-metalate shift approach for vinylidene homologation of organoboronates, where a Pd-allyl complex activates the ate-complex, promoting a 1,2-metalate migration coupled with β-hydride elimination to yield 1,1-disubstituted alkenyl boronic esters (Figure 4).^53^ Aryl, primary, and secondary alkyl boronic esters are well tolerated. In the same year, Aggarwal reported a vinylidene homologation approach using lithiated epoxysilanes, enabling stereospecific insertion of the sp^3^-carbon unit followed by a *syn*-Peterson elimination.^54^ Combining with the reagent-controlled stereospecific homologation (see subsection 5 “**Enantioselective homologation**”), this method was employed in an iterative fashion to access complex polyenes, including a short synthesis of the proposed structure of machillene.

**Figure 4:**
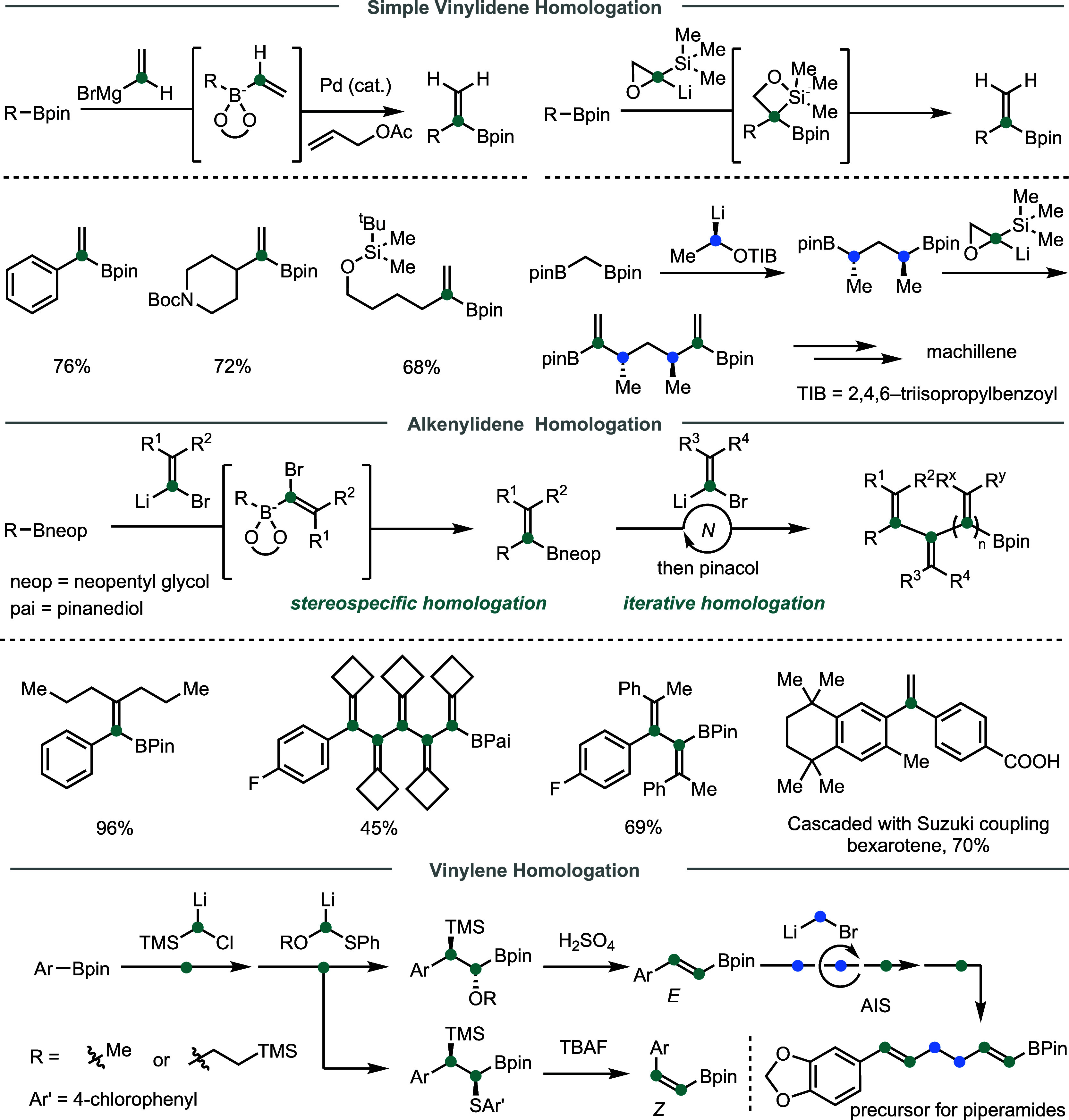
Development of sp^2^-carbon insertions.

Recently, Liu and Dong developed an alkenylidene homologation protocol, in which a boronic ester undergoes stereospecific homologation with 1-bromo-1-lithioalkene via a strain-released S_N_V mechanism.^55^ Besides monoalkenylidene insertion, this approach also enables programmable incorporation of multiple alkenylidene units, facilitating the synthesis of stereodefined cross-conjugated polyenes that are challenging to prepare otherwise. Further coupling with Suzuki reactions simplifies the assembly of bioactive compounds bearing multisubstituted alkenes, such as, bexarotene. The same team also developed a diastereoselective vinylene homologation strategy,^56^ which involves consecutive insertion of silyl- and alkoxy-substituted carbenoids followed by Peterson-type elimination. Both the *Z* and *E* alkenyl boronates can be selectively obtained under complementary conditions. To demonstrate the AIS concept, combining this vinylene homologation with methylene homologation provides a unified iterative approach that efficiently constructs the polyene backbone of piperamides.

#### 2.1.3 Iterative Construction of Cyclic Structures

Ring structures are commonly found in functional organic molecules; however, the Matteson reaction has been rarely explored to create rings.^57^ In 1999, Matteson reported a diastereoselective synthesis of cyclobutanes via intramolecular boronate homologation (Figure 5). The deprotonated nitrile served as a nucleophile to attack the α-chloroboronate to furnish ring closure with high diastereoselectivity. However, extension of this method to form cyclopropanes encountered difficulties to control diastereoselectivity.^58^ Recently, an improved annulation strategy was reported by the Dong group.^59^ By employing *tert*-butyl ester as the annulation handle, along with LTMP as the base and a catalytic amount of MgBr_2_, a diverse range of carbocycles (3–6 membered rings) with multiple stereocenters and diverse substituents can be constructed. Besides esters, other electron-withdrawing groups (EWGs) can also mediate similar annulations. In addition, through iterative homologation and annulation, all-carbon spirocycles and double spirocycles can be constructed. Thus, this annulation strategy could serve as a versatile platform for the programmable synthesis of cyclic architectures.

**Figure 5:**
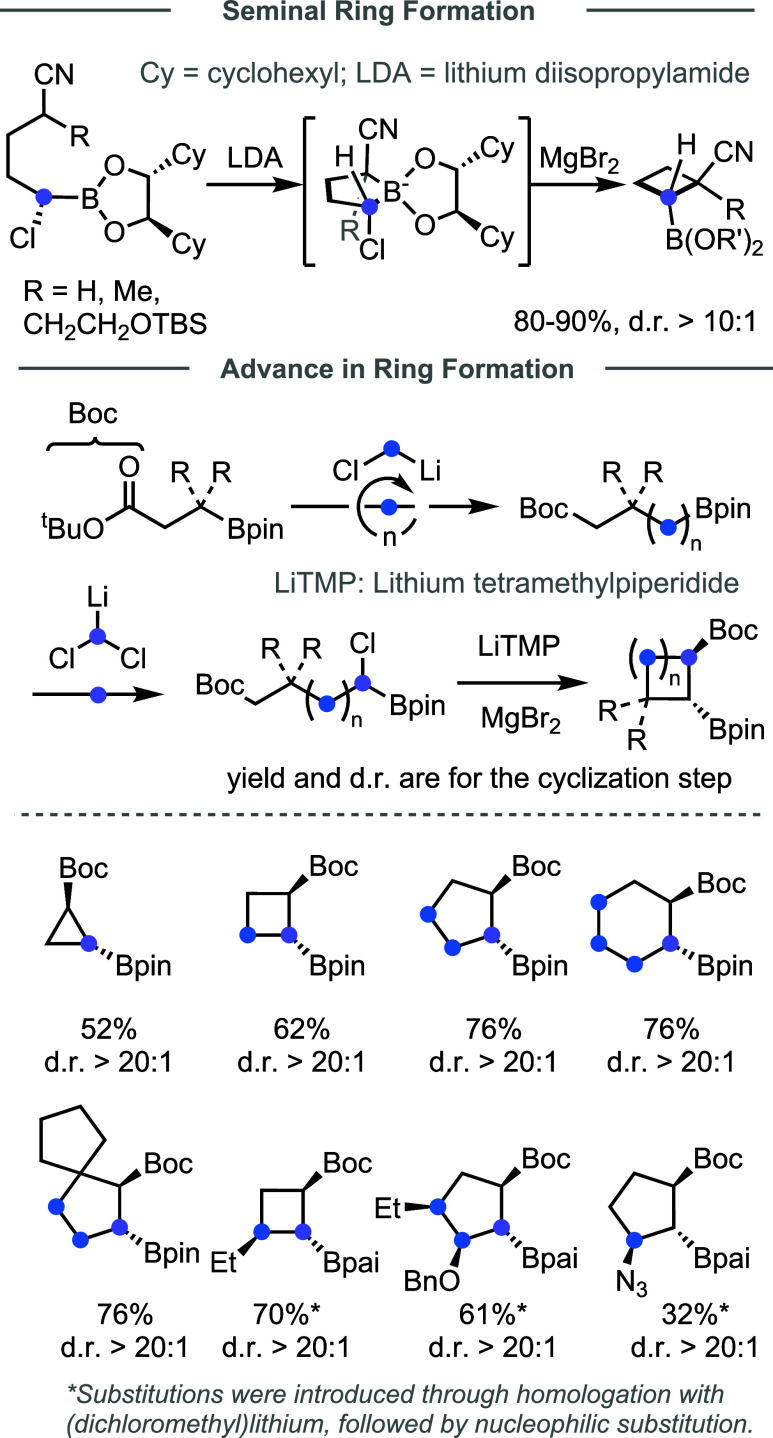
Development of homologative cyclization.

#### 2.1.4 Thermostable Carbenoids

Most of the carbenoid reagents employed in Matteson-type reactions require the use of pyrophoric bases for their preparation. In addition, due to their high instability, highly cryogenic conditions are typically needed during the generation and reaction of these carbenoids. From the prospective of practicality of the AIS strategy, it would be more attractive to develop boron homologation reactions under milder conditions by avoiding pyrophoric bases and cryogenic conditions.

While there remains no general solution to this challenge, a recent study from the Dong group offers some promises, in which a new class of carbenoids featuring sulfinate as the LG was discovered (Figure 6).^60^ These carbenoids exhibit greater thermostability. For example, they allow direct insertion of oxygen- and nitrogen-substituted methylenes into oxaborolidines at 0 °C or *even completely at room temperature*. Notably, the electron-withdrawing ability of sulfinates allowed less reactive bases, such as LiHMDS or LDA to be suitable for generating these carbenoids. As a result, FGs that are sensitive to strong bases or nucleophiles, including aryl iodides, amides, esters, and acidic protons adjacent to esters, were tolerated under the reaction conditions.

**Figure 6:**
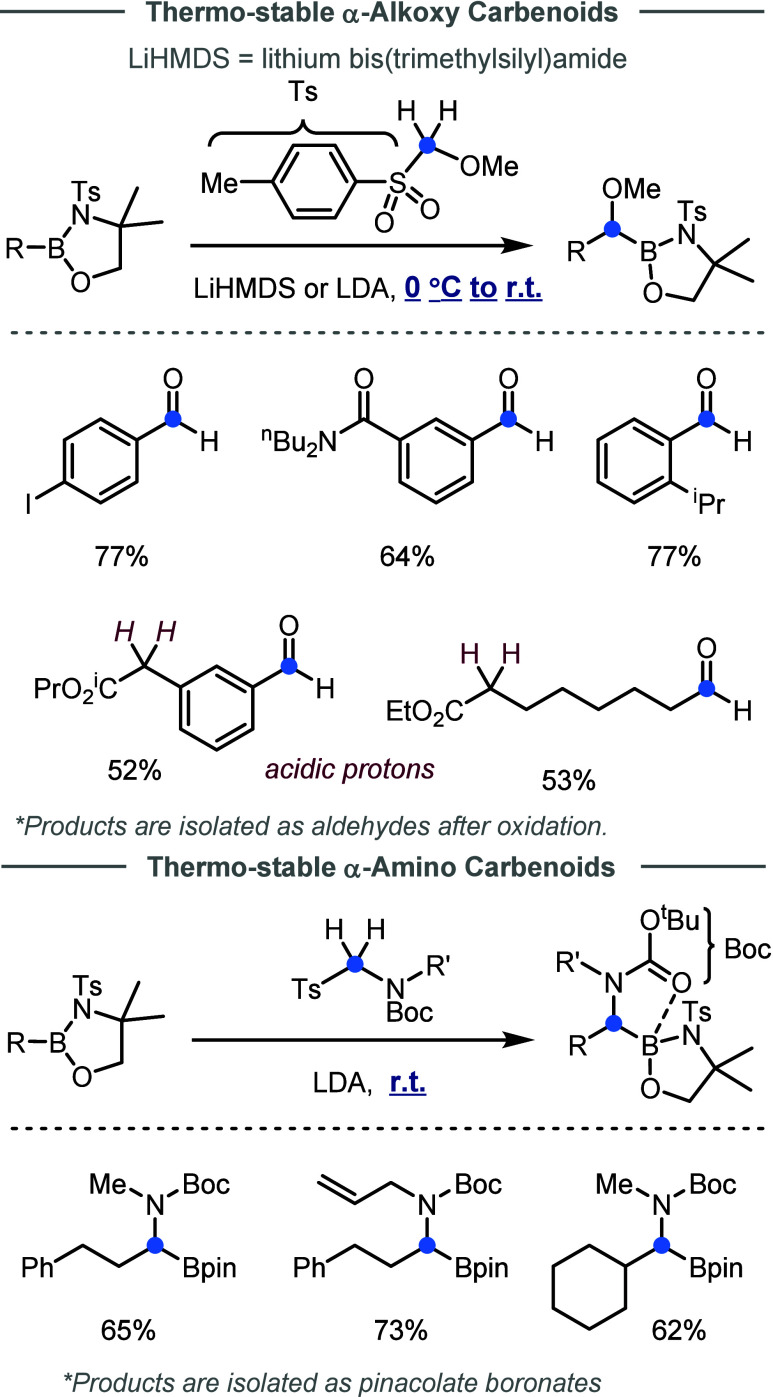
Development of thermostable carbenoids.

#### 2.1.5 Enantioselective Homologation

Being capable of preparing chiral molecules with high enantiopurities is certainly an important objective for AIS. In the past few decades, significant advances have been made in asymmetric boron homologation reactions. To date, three general strategies have been developed: (1) desymmetritive homologation with achiral dichloromethyllithium,^61–63^ (2) stereospecific homologation with enantioenriched carbenoids,^64–66^ and (3) enantioconvergent homologation with racemic carbenoids.^67,68^

The desymmetritive homologation strategy (Figure 7) employs achiral carbenoid LiCHCl_2_ to install a chloromethylene unit into a boronic ester in a stereoselective fashion, followed by replacing the chloro group by a nucleophile with stereoinversion to afford enantioenriched boronates. In Matteson’s original approach, the diastereoselective 1,2-migration is governed by chiral auxiliaries, including (+)-pinanediol, (1*R*,2*R*)-1,2-dicyclohexylethane-1,2-diol, and (*S*,*S*)-diisopropylethane-diol, which induce high levels of stereocontrol.^69,70^ In Jacobson’s approach, a chiral lithium catalyst^71^ is employed to promote the enantioselective 1,2-migration with alkyl boronates. Using the desymmetritive homologation approach, two steps are needed to install substituted carbenoids. The alternative stereospecific strategy relies on the chiral carbenoid reagents’ pre-existing stereochemical information to control the outcomes of the homologation process. Blakemore employed highly enantioenriched α-chloro sulfoxides as the carbenoid precursor (prepared by Yamakawa chlorination),^72–75^ which then undergo a stereoretentive sulfoxide–metal exchange to generate the enantioenriched carbenoid. Subsequently, through ate complex formation with boronates and 1,2-metalate rearrangement, the chirality of the carbenoid is relayed to the homologation product. While this approach is highly effective, erosion of enantiopurity during the preparation of these carbenoids often occurs, caused by epimerization of the sulfoxide precursors. By contrast, the Hoppe-type carbenoids (e.g., lithiated carbamates or benzoates) exhibit greater configurational stability,^76^ and the enantioselective deprotonation enabled by sparteine afforded highly enantioenriched (>99% enantiomeric excess) carbenoids. Extensive efforts by Aggarwal have elegantly demonstrated the utilities of these carbenoids in various asymmetric boron homologation reactions.^77^ Besides tertiary stereocenters, quaternary ones can also be efficiently constructed. Moreover, iterative homologation to construct complex linear molecules with multiple stereocenters^34–40^ and asymmetric insertion of a silyl-substituted methylene were also realized.^78^

**Figure 7:**
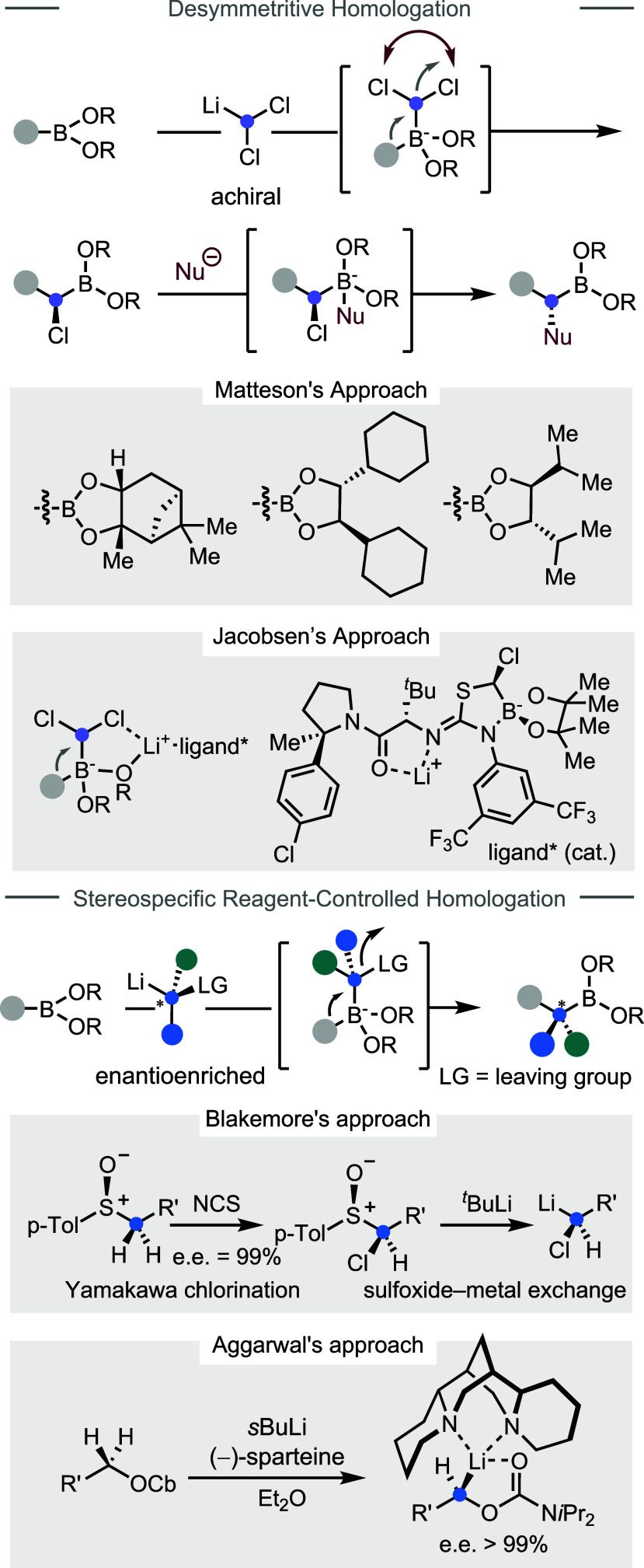
Desymmetritive and stereospecific homologations of boronates.

Recently, the Dong group reported an enantioconvergent approach for a general boron homologation using various racemic carbenoids (Figure 8).^68^ This approach took advantage of a new class of chiral boronic compounds, namely, oxazaborolidines, which exhibit excellent stereochemical control and enhanced reactivity compared to boronates. These oxazaborolidines are derived from inexpensive amino acids. Readily available racemic carbenoids can be directly used. A key feature is that fast racemization of carbenoids facilitates a dynamic kinetic resolution^67,79,80^ in the presence of the chiral boron substrate. Besides carbon-substituted carbenoids, by adjusting the structures of the chiral auxiliaries, oxygen-, nitrogen-, sulfur-, and silicon-substituted carbenoids can all be inserted to yield the corresponding homologation products with high enantioselectivity. In addition, the programmable synthesis of contiguous stereocenters through the iterative coupling was also achieved in excellent stereoselectivity. For example, the synthesis of a key intermediate for fumonisin B1, containing a triad of contiguous stereocenters, was achieved through consecutive enantioconvergent homologations in which only one column purification was employed at the end of the synthesis.

**Figure 8:**
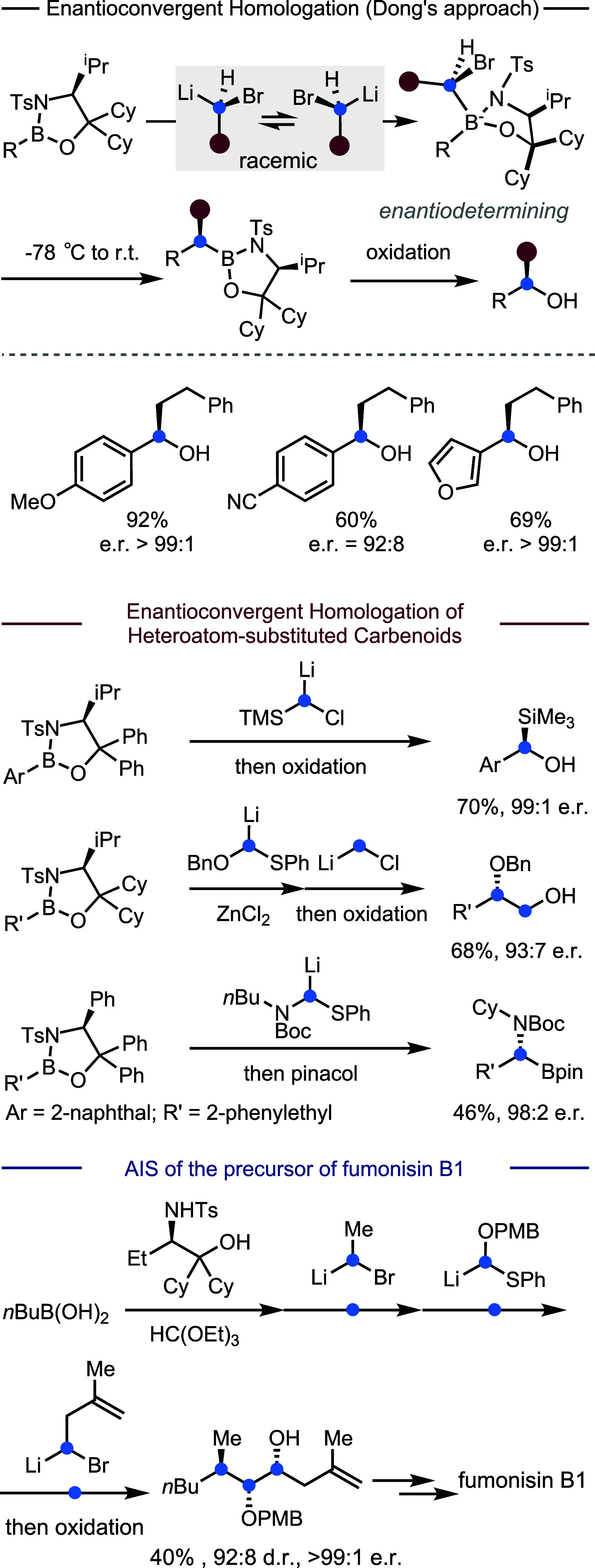
Development of enantioconvergent homologation of boronic compounds.

## 3 OUTLOOK

Tremendous progress has been achieved toward programmable syntheses of fine organic molecules based on the AIS logic. However, substantial limitations or constraints remain to be addressed at this stage in order to allow AIS to be a suitable approach for automated synthesis (Figure 9). For example, regarding insertion nitrogen atoms to molecular backbones, couplings with aryl- or tertiary alkyl-substituted nitrenoids have not been achieved.^50^ In addition, other heteroatom BBs, such as sulfur-based ones, have not been explored in boron homologation, which, if successful, would enhance skeletal diversity of the compounds that can be made by AIS. On the other hand, while insertion of simple vinylidene has been demonstrated, access to more substituted alkenes using the AIS strategy has not been investigated yet.^55^ Moreover, a broader question is how to access aromatic compounds using AIS? Without using large aromatic BBs, identification of suitable atomic-scale BBs that can assemble into aromatic structures is expected to be a critical and nontrivial challenge. Furthermore, given the importance of sp-hybridized carbons in functional organic molecules, it would be valuable to allow the installation of triple bonds via boron homologation.

**Figure 9:**
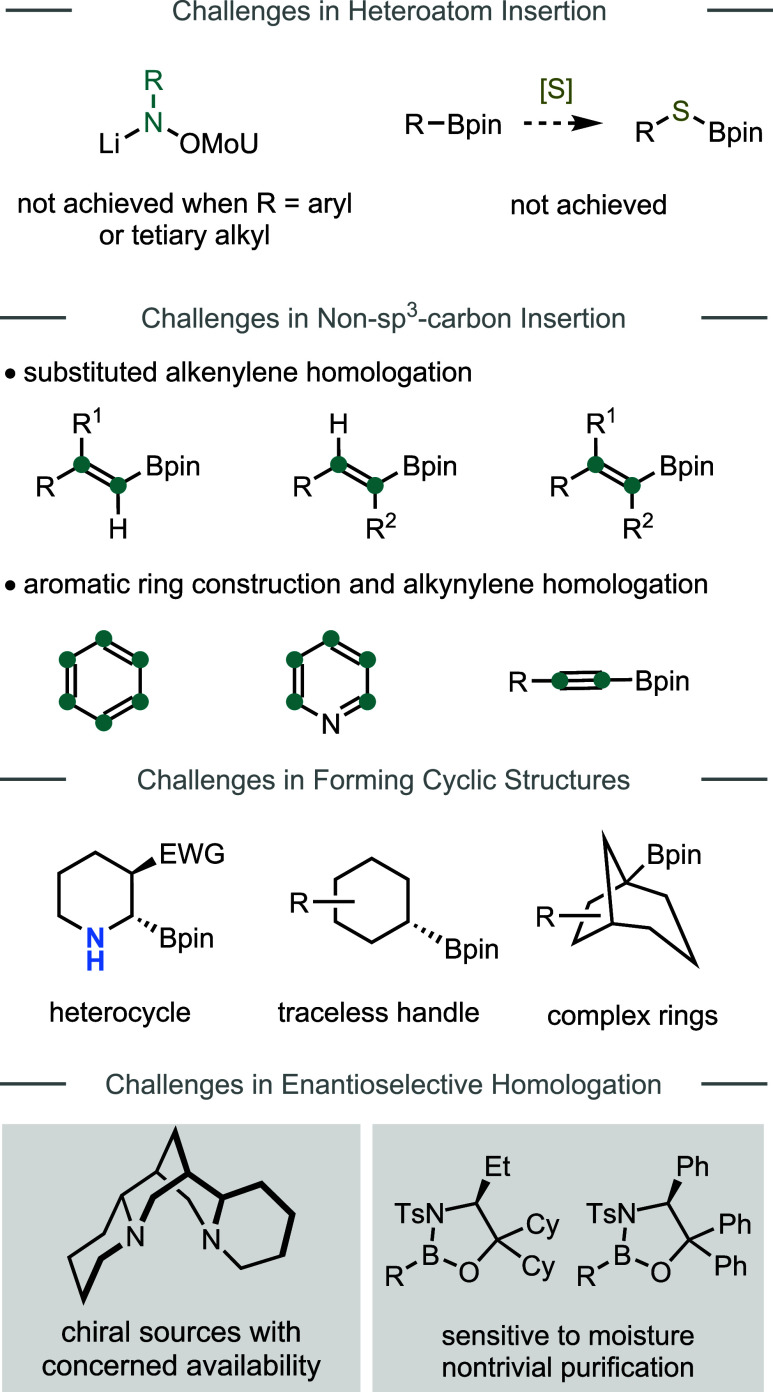
Key challenges and limitations in advanced boron homologation chemistry.

Regarding the ring forming strategy, the current method relies on the use of an EWG that may not be needed in the target molecules, and only carbocycles are constructed so far.^57,59^ The development of more general annulation approaches that can construct both carbo- and heterocycles would be essential for broader applications to more complex targets.^81–83^ Concerning the thermostable carbenoids, the current successful ones contain a methoxy or amino group, which can enhance the leaving ability of the sulfinate group.^60^ Better understanding of the mechanism and further modification of structures of the electron-withdrawing LGs are likely necessary to extend the scope for introducing more types of thermostable carbenoids.

Enantioselective boron homologation is perhaps the most developed area compared to other categories. It could be fair to state that the current desymmetritive, stereospecific, and enantioconvergent approaches all have their advantages (mentioned earlier) but also some constraints. The desymmetritive approach needs a two-step operation, and not all types of FGs can be introduced as nucleophiles.^29–33^ While the stereospecific strategy has been shown to be highly powerful for constructing continuous stereocenters, insertion of heteroatom-substituted carbenoids remains challenging. In addition, the availability and turnability of the chiral sources used, i.e., sparteine, could cause inconvenience.^84^ Moreover, the use of stoichiometric tin reagents in some cases could be a concern.^85,86^ While Dong’s enantioconvergent approach can introduce various heteroatom-substituted carbenoids, construction of diverse quaternary carbon centers has not been achieved yet.^68^ In addition, compared to boronates, oxazaborolidines used in the enantioconvergent approach are more moisture sensitive, thus challenging to be directly purified.^68^ Finally, for all chiral auxiliary-based strategies, efficient auxiliary switch protocols must be developed in order to meet the demand for programmable syntheses.

Almost all of the aforementioned limitations are in principle addressable through further development, which could offer opportunities for future directions. In addition to the areas that have been investigated, a number of unexplored questions should also be considered and addressed in order to realize a future automated synthesizer based on the AIS logic:

### 3.1 How Can Branched Structures Be Accessed?

Branched structures are highly prevalent in organic compounds. However, the existing Matteson-type homologations almost solely focus on the synthesis of linear compounds. It would be critical to design suitable bifunctional BBs for introducing a branching point during homologation, which can lead to compounds containing two or more branched chains.

### 3.2 How Can Sensitive FGs Be Introduced?

Generally, three types of FGs are incompatible with the Matteson-type reactions: a) FGs that are more electrophilic than boronates, such as ketones, aldehydes, nitro, and some ester groups, as they preferentially undergo irreversible addition reactions with carbenoid reagents; b) FGs containing highly acidic protons, such as carboxylic acids and alcohols, which can directly quench carbenoid reagents; and c) strongly Lewis basic FGs, such as amines, which can potentially inhibit the reactivity of the boron center.^87,88^ One can imagine that these FGs should be introduced either in their protected forms or in the last stage of the synthesis.

### 3.3 How Can We Simplify the Purification Process?

Although high yields can be achieved with simple model substrates, moderate or even low conversions are often encountered when the substrates become more complex. The accumulation of side products or unreacted substrates can lead to a complex mixture. Thus, the challenge of purification using the AIS logic cannot be overlooked, and it is important to minimize chromatography separations. As such, besides continuing to improve the reaction efficiency, it is anticipated that the development of new and efficient separation methods tailored to the boron homologation reaction would be crucial.

### 3.4 How Can the Optimal Synthetic Route Be Determined?

For a given target molecule, numerous sites could be the starting point of the synthesis, and a large number of different synthetic routes could be possible based on the AIS logic. It is almost certain that identification of the optimal synthetic route would benefit from modern machine learning technologies, which are expected to rely on the knowledge gained on various boron homologation reactions that have been developed and are to be developed.

### 3.5 How to Minimize Economic, Environmental, and Time Costs?

Owing to the linear operational feature, an impression of the AIS logic is that a large number of steps would be needed when preparing a complex molecule. This could result in higher expenses, more waste, and more time compared to the conventional FGFA approaches. Thus, it would be imperative to enhance the efficiency of the homologation chemistry to shorten the time of operation, to minimize the use of excess reagents, and to simplify purification processes. When each coupling event becomes rapid and high yielding without frequent purification, they would be nearly equivalent to an operation in a conventional step that typically contains multiple operations. In addition, the use of more unified coupling approaches would also reduce the complexity of the wastes generated, therefore, easing the recycling or disposal processes.

Aspired by the promising preliminary achievements summarized in this article, the advantages of using simple versatile atomic-scale building blocks (BBs) and unified assembly methods could make AIS an appealing platform for developing compact and fully automated synthesizers without relying on unlimited kinds of reagents or custom-made BBs.

## Conclusion

Compared with the well-established FGFA approaches, the development of the AIS logic for organic synthesis is still in its infant stage. Aspired by the promising preliminary achievements summarized in this review, the advantages of using simple versatile atomic-scale BBs and unified assembly methods could make AIS an appealing platform for developing compact and fully automated synthesizers without relying on unlimited kinds of reagents or custom-made BBs. While we are excited and optimistic about the blueprint, a number of significant challenges and uncertainties would require systematic investigations and innovative designs to address. The insights and knowledge gained during these explorations would lay the foundation of the AIS logic. It is our hope that this Outlook article could provide some inspiration to those who are interested in the topic of automated organic synthesis.
